# Reflection on clinical and methodological issues in rare disease clinical trials.

**DOI:** 10.1186/s13023-025-03805-1

**Published:** 2025-06-05

**Authors:** Johan Verbeeck, Martin Geroldinger, Joakim Nyberg, Konstantin E. Thiel, Andrew C. Hooker, Arne C. Bathke, Johann W. Bauer, Geert Molenberghs, Martin Laimer, Georg Zimmermann

**Affiliations:** 1 I-BioStat, Hasselt University, Martelarenlaan 4, 3500 Hasselt, Belgium; 2 Team Biostatistics and Big Medical Data, IDA Lab Salzburg, Paracelsus Medical University, Strubergasse 21, 5020 Salzburg, Austria; 3 Department of Neurology, Christian Doppler Medical Centre, Paracelsus Medical University, Ignaz-Harrer Straße 79, 5020 Salzburg, Austria; 4 Department of Pharmacy, Uppsala University, 75124 Uppsala, Sweden; 5 Intelligent Data Analytics (IDA) Lab Salzburg, Department of Artificial Intelligence and Human Interfaces, University of Salzburg, Kapitelgasse 4/6, 5020 Salzburg, Austria; 6 Department of Dermatology and Allergology, Paracelsus Medical University, Ignaz-Harrer Straße 79, 5020 Salzburg, Austria; 7 I-BioStat, KU Leuven, Kapucijnenvoer 35, 3000 Leuven, Belgium; 8 Research Programme Biomedical Data Science, Paracelsus Medical University, Strubergasse 21, 5020 Salzburg, Austria; 9 Department for Artificial Intelligence and Human Interfaces, Paris Lodron University, Jakob-Haringer-Strasse 2, 5020 Salzburg, Austria

**Keywords:** Rare disease, Epidermolysis bullosa, Endpoints, Non-parametric marginal model, Generalized pairwise comparisons, Generalized estimating equations, Model averaging, Visual analogue scale

## Abstract

Rare diseases present critical challenges to healthcare systems, patients, and caregivers due to their low prevalence and unique characteristics. Designing clinical trials and developing statistical methodologies for evaluating interventions in rare diseases face several challenges. The “EBStatMax” project, part of the European Joint Programme on Rare Diseases’ Demonstration Projects, aimed to address one of these challenges, namely: designing and analyzing longitudinal cross-over data in rare diseases, like Epidermolysis bullosa simplex (EBS). Although the main findings of the project have been published elsewhere, this manuscript reflects on additional hurdles encountered during the project, particularly regarding outcomes and methodological considerations. It explores issues surrounding outcome measurement, statistical methodology, and clinical considerations, emphasizing their broader relevance to methodological advancements in rare disease research beyond this specific case. This manuscript highlights the critical role of international collaboration in rare disease research to enhance evidence quality and aims to inspire further advancements in the field.

## Introduction

While the definition of a rare disease varies by prevalence, it is generally accepted that the number of rare diseases is substantial [1, 2]. Collectively, they impose a significant burden on healthcare systems, societies, and most importantly, on the patients and their caregivers [3, 4]. Consequently, numerous strategies, initiatives, funding programs, and projects have been launched, with the aim of improving the diagnosis, prognosis, and treatment of patients with rare diseases.

In rare diseases, as in other fields of medicine, randomized controlled clinical trials are regarded as the gold standard for establishing the efficacy and safety of treatments. However, compared to other domains of clinical research and pharmaceutical development, rare diseases present particular challenges. These include, for example, limited patient populations, disease heterogeneity, and the need to identify and validate appropriate endpoints, among others. While some of these issues have been addressed by methodological experts and statisticians in a number of EU-funded projects [5–7], many unresolved problems remain, necessitating further investigation. Additionally, the innovative statistical approaches developed through these projects are often underappreciated and underutilized. To facilitate understanding of the often complex innovative methodological approaches and to foster the translation into practice, three so-called “Demonstration Projects” have been funded in 2020 by the European Joint Programme on Rare Diseases [8].

One of those demonstration projects,“EBStatMax” [9], aimed to:

1. Conduct systematic empirical comparisons, by means of simulations, of existing innovative statistical methods for analyzing longitudinally collected data from rare disease clinical trials;
2. Provide educational materials and guidance to encourage adoption of these methods by applied researchers and other stakeholders in rare diseases;
3. Implement the most promising statistical approaches in accessible, user-friendly software tools.

The project focused on a specific clinical case, but the main findings are applicable to other rare diseases with similar study design characteristics. The chosen case was a single cross-over clinical trial assessing the efficacy of Diacerein cream versus placebo in patients with Epidermolysis bullosa simplex (EBS) on blister counts, pain, pruritus, and quality of life (QoL) [10].

Epidermolysis bullosa (EB) is a group of rare inherited disorders characterized by fragile epithelial-lined tissues and surfaces, particularly the skin [11]. EBS is the most common EB subtype, which is marked by the formation of blisters under low mechanical stress, causing substantial burden during daily activities for affected individuals.

In the case trial, outcomes were assessed at four time points: baseline, 2 weeks, end-of-treatment at 4 weeks and follow-up at 3 months. However, the original analysis focused solely on the end-of-treatment time point for assessing the primary endpoint [10]. The EBStatMax project addressed this limitation by identifying and comparing statistical methods that leverage the available longitudinal data, both for the blister count outcome [12] as well as for pain and pruritus [13]. This resulted ultimately in practical recommendations for rare disease cross-over trials with missing data on the identified methods: non-parametric marginal models, generalized pairwise comparisons (GPC), generalized estimating equations (GEE) models, and model averaging[14]. In addition, the project explored optimal design for longitudinal cross-over trials [15], as well as how to combine diverse outcomes, such as blister counts and QoL measurements in a single, comprehensive analysis [16]. Several of these methodologies have also been implemented in user-friendly software.

The interdisciplinary composition of the project’s consortium, which included expertise in biostatistics as well as complementary clinical and methodological research, led to the emergence of several valuable methodological questions. The aim of this manuscript is to provide a comprehensive summary of these reflections and discussions, focusing on outcome measurements (Sect. Reflection on outcome measurements), statistical methodology (Sect. Reflection on statistical methodology) and clinical aspects (Sect. Reflection on clinical and patient-relevant aspects). By highlighting these topics, we hope to foster and guide future research. This discussion is primarily targeted at statisticians and methodological researchers, although it would also be valuable to other stakeholders and practitioners within the rare-diseases community. Addressing these reflections is important because they have the potential to shape priorities for funding agencies, stimulate innovative methodological approaches among applied biostatisticians, and ultimately improve the quality of research and care in rare diseases.

## Reflection on outcome measurements

### Number of blister lesions

The primary outcome in the cross-over trial “use case” was the number of blister lesions counted by clinicians on a pre-specified area of the skin repeatedly over time [10]. However, using blister counts over time to measure treatment efficacy is subject to variability from multiple sources. First, blisters are not always clearly delineated, leading to uncertainty about whether a lesion represents one or multiple blisters, when assessed at a particular (single) time point. This results in non-negligible inter- and intra-rater variability in outcome assessment. Secondly, some blisters heal spontaneously, potentially causing an overestimation of the treatment effect. These uncertainties in the blister count outcome were circumvented by the dichotomization of the blister count in the case trial. A reduction of more than 40% in the number of blisters was considered a treatment effect, while reductions of 40% or less were not. This dichotomization aimed to reduce the impact of uncertainty in the blister count on the treatment assessment. However, dichotomization can result in a loss of information and, consequently, a loss of power to detect treatment effects. For example, a simulation study using one of the most powerful tests, the unmatched prioritized GPC, showed a decrease in power to detect treatment effects from 53% to 10% when shifting from raw to dichotomized blister counts [12]. Needless to say that in small-sample trials, maximizing the use of the available information and minimizing loss of information are critical considerations.

To optimize the use of available information in small sample trials, while accounting for non-negligible variability in outcome measurements, further research is required. For example, incorporating a reference baseline period into the study design could help address uncertainties related to blister occurrence and spontaneous healing. Before the first administration of the trial drug, blister dynamics could be monitored over a sufficiently long baseline period (for example, 4–6 weeks). Depending on the methodology, the average blister count – and possibly its variance – during this baseline period could serve as a covariate in modeling, a standardization factor, or even as part of longitudinal modeling approaches. Interestingly, in GPC, the uncertainty in blister counts can be managed by introducing a threshold in pairwise comparisons. In this context, a subject’s blister count is considered better than another only if their (standardized) blister count differs by at least the natural variability observed during the baseline period. This latter strategy is a way of analyzing the data which is somehow in-between the raw blister counts, which fail to account for uncertainty, and dichotomized counts, which lacks power, thereby balancing uncertainty and power.

Given these challenges, it is important to critically evaluate how different statistical methods utilize available information. For example, the semi-parametric GEE-type models and the parametric models in the model averaging method used in the EBStatMax project analyze the longitudinal profile of the probability of observing certain blister counts. These approaches implicitly account for some measurement uncertainty. On the other hand, non-parametric marginal models and the GPC method rely on ranks (or the equivalent pairwise comparisons), which focus on the relative position of the counts rather than their absolute differences. Relative measures may be less sensitive to the blister-count uncertainty, especially when the counts are standardized using baseline counts. Nevertheless, a sufficiently flexible (semi-)parametric approach should be able to accommodate both absolute and relative differences. Further research may focus on enhancing (semi-)parametric models to better incorporate measurement error, for example, in a residual term.

### Pain and pruritus: ordinal or metric visual analogue scale (VAS)

In the case trial, both pain and pruritus were measured on a visual analog scale (VAS). Patients answered the question “How do you perceive your level of pain/pruritus?”, by marking a point on a continuous scale ranging from 0 cm (“absence of pain/pruritus”) to 10 cm (“most intense pain/pruritus imaginable”), with an increment of 0.5 cm, While this looks like a metric scale, it is recommended to treat VAS scores as ordinal because differences between scores cannot consistently be interpreted in a meaningful way [17]. For instance, the difference between “3” and “1” has clinically and intra-individually a different connotation than the difference between “8” and “6”, although the absolute difference remains the same. Additionally, although the short-term reliability of VAS scores for pain (within 24 h) is well-documented, their long-term reliability remains questionable [18].

Although modeling ordinal longitudinal data is relatively straightforward, the ordinal-metric nature of VAS scores poses unique challenges for their longitudinal evaluation. Analyzing absolute VAS values assumes long-term reliability of the scores, while baseline-corrected absolute differences rely on the assumption that differences carry the same significance for all individuals throughout the range of values. Similarly, relative differences compared to baseline assume long-term reliability of the scores and require a nonzero baseline score. Although relative differences have been recommended [19], the EBStatMax project focused solely on absolute values and baseline-corrected absolute differences, since many baseline VAS scores were zero [13]. Note that the change from baseline may be in contradiction to the ordinal nature of VAS variables. Alternatively, dichotomizing VAS scores – such as determining whether the score decreases by at least 30% – would be more robust against fluctuations due to objectivity, but may lose granularity of the data.

In EBStatMax we prioritized non-parametric methods that rely on the order of values. However, this approach also has challenges related to the meaningful analysis and interpretation of VAS scores. Indeed, given the intra- and inter-subject variability of VAS scores, they may have less value for comparing VAS scores across groups of individuals at one specific time point. Comparison of VAS scores between subjects may be possible, but it would require a large amount of data or subjects to keep the variability under control. Precisely this is an issue in the context of rare diseases. A potential solution may be to compare VAS scores with baseline scores and define the following categories:

- improvement (when the difference exceeds a clinically or subjectively relevant threshold);
- stable (when the absolute difference is less than a threshold);
- worsening (when the difference is smaller than the complement of a relevant threshold).

However, dividing the VAS score into these categories may lead to loss of information and consequently loss of power. Apart from that, it might be difficult to establish a unified definition of the categories that is based on solid, thorough reasoning.

Future research should focus on identifying the most appropriate methods for analyzing the metric-ordinal VAS scores (see Sect. Methodologies for VAS scores) and in identifying the smallest reliably detectable change and the minimally important difference at which patients experience a chance in symptoms or disease burden. Additionally, it is worth exploring whether the burden of disease might be better captured by a quality of life (QoL) scale, which could provide a more comprehensive measure than separate VAS scores for pain and pruritus.

### Longitudinal data and multivariate outcomes

In small-sample trials, where the number of participants is limited, it is of the utmost importance to optimize the use of available information when assessing a treatment. One strategy to maximize information is to evaluate longitudinal measurements of the clinical outcome of interest, provided such data is available. However, collecting measurements in EBS are often invasive, including more extensive bandage and dressing changes, causing discomfort for the patients. This may impair the willingness for patients to participate in clinical trials, the compliance and adherence to the trial schedule and the recruitment. Another strategy to maximize information is to incorporate multiple outcomes into the analysis. Rare diseases are often multifaceted, making it challenging – and sometimes inadequate – to select a single clinically relevant endpoint that fully captures the therapeutic benefits of a treatment. Moreover, patient-centered outcomes, such as QoL assessments, are increasingly recognized as essential for evaluation of treatments [16, 20, 21] and should be considered alongside more traditional outcomes.

In the EBS trial, both the number of blisters and QoL were assessed, reflecting the significant impact of the disease on daily activities for patients. However, combining outcomes of different data type in a single analysis presents specific challenges. For instance, multiple testing procedures often lead to a decrease in power, while semi-parametric and parametric modeling approaches may be difficult to apply in small-sample settings. GPC and non-parametric multivariate methods [22, 23] have been shown to be effective and easy to use methods to assess a treatment in a small sample with multiple outcomes of varying data type, such as, blisters (as a count or binary outcome) and QoL ( as an ordinal outcome) [16].

Despite their promise, the potential of these non-parametric methods for analyzing multiple outcomes warrants further exploration. Comparisons with other methodologies are needed, including, but not limited to:

- Parametric combined models with split sample [24–26] or pseudo-likelihood inference [25–27]
- Item response theory [28]
- The O’Brien methods [29] and its variations [30]
- Non-parametric and semi-parametric MANOVA [31]
- Multiple testing procedures [32]

In addition, the GPC method should be expanded to handle both longitudinal and multiple outcomes simultaneously, which would further enhance their utility in complex trial designs.

Finally, endpoints and methodologies that allow for simultaneous assessment of blister counts, the type of blisters (healing or non-healing), pruritus and pain should be further developed.

### Data-generating mechanism for simulation-based method comparison

In the EBStatMax project, several statistical methodologies were compared in terms of their potential to evaluate longitudinal cross-over information in small sample trials. This evaluation was based on their ability to control type I error, the power to detect a treatment effect and handling of missing data within a simulation study. This study permuted the original observations in the case trial to remove any treatment effects and subsequently inserted artificial effects based on clinical reasoning [12, 13, 16]. This approach was chosen because it primarily relies on observed data, omitting the requirement of making several (distributional) assumptions when simulating data in a traditional way by drawing observations from distributions. Alternatively, a mixed-effects model, such as the one developed in Verbeeck et al. [12] could serve as a data-generating mechanism. Of course, any mixed-effects model is subject to assumptions, which may be misspecified.

A persistent challenge in comparing statistical methodologies lies in creating simulation scenarios that do not inadvertently favor one method over another. For instance, in our simulations, the GPC methodology might have been advantaged due to the prioritization of certain time points. Conversely, using a mixed-effects model as the data-generating mechanism could inherently favor parametric modeling approaches.

Further research is needed to evaluate the utility of several data-generating mechanisms. Regardless of the approach, simulated scenarios should be grounded in clinical expert knowledge, derived from sources such as registries, trials or clinical experience. In addition, simulations should explore several realistic scenarios and assess the impact of various forms of misspecification through sensitivity analyses.

## Reflection on statistical methodology

### Missing data

For some methods employed in the EBStatMax project, strategies to handle missing data are well-established [33]. For example, likelihood-based or Bayesian methods ensure validity under the assumption of missingness at random (MAR), meaning the missingness mechanism can depend on covariates and observed outcomes, but not on unobserved outcomes. However, GEE, which lacks a likelihood foundation, requires particular care. Options include using weighted GEE (WGEE), or pre-processing GEE with multiple imputation (MI; [34]). Multiple imputation is a versatile technique, applicable not only to GEE, but also to any method without a likelihood or Bayesian framework, including the non-parametric methods discussed in this text [35]. Because missingness not at random (MNAR) – where missingness also depends on unobserved outcomes – cannot be ruled out based on observed data, sensitivity analyses are crucial. These analyses assess the impact of unverifiable assumptions about the missing data mechanism on key inferences, such as treatment effects. Sensitivity analyses can be conducted using multiple imputation based methods [36].

At the time of the EBStatMax project, the non-parametric marginal models could only accommodate fully observed longitudinal profiles. Consequently, profiles with missing data had to be excluded from the analysis, which is suboptimal in rare disease trials, where data is already scarce. However, an extension of these models has since been published, allowing for missing data in a clustered-data setting [37]. Future research should examine how this adapted approach handles different missingness mechanisms, in particular MAR and MNAR. A comparison with an MI-based approach would also be highly valuable.

In the unmatched GPC, initial studies on the missingness mechanisms have been conducted [38], but further exploration is needed. This is true for matched GPC, whether used independently or in conjunction with MI.

### Covariate adjustment for non-parametric methods

An issue commonly encountered in non-parametric statistical methodology is the limited ability to correct for covariates beyond treatment. The non-parametric methods evaluated in the EBStatMax project are no exception, although the non-parametric marginal models allow for a stratification factor for repeated measures, alongside the treatment. Likewise, a GPC analysis can be stratified in pre-defined subgroups [39]. However, dividing an already small sample into even smaller strata may become prohibitive in some rare-disease clinical trials. Although covariate adjustment was not necessary for the analyses in the EBStatMax project, it was identified as a limitation of the non-parametric methodologies.

To ensure wider applicability, further research should focus on extending the non-parametric marginal models to incorporate covariate adjustment, which may be achieved using ideas from the univariate non-parametric ANCOVA models [40] and the semi-parametric repeated measures ANCOVA models [41]. In contrast, semi-parametric GPC regression models have already been suggested for univariate outcomes [42], which have been extended to multivariate outcomes [43]. However, these models depend on asymptotic assumptions and further research is needed to study their performance in small sample settings and, if necessary, to propose improvements.

### Methodologies for VAS scores

When assessing treatment effects using a VAS score, as performed for both pain and pruritus in the EBStatMax project, the outcome is often treated as ordinal. In small samples, where fitting full likelihood models, such as (continuous) ordinal regression models [17, 44] is challenging, longitudinal ordinal outcomes can be modeled marginally using GEE-type ordinal logistic models. These models do not explicitly describe the association structure, but rather replace it with a potentially misspecified working assumption, while still yielding valid inferences. However, several issues were encountered when modeling the VAS score with these GEE-type models. First, since the VAS score is measured with an accuracy of 0.5 cm (see Sect. Pain and pruritus: ordinal or metric visual analogue scale (VAS)), a transformation of the outcomes is needed. For example, multiplying the VAS scores by 2 yields an ordinal outcome, now ranging from 0–20, which can be analyzed using an ordinal logistic model. Second, small sample bias corrections are necessary when the variance exhibits heteroskedasticity over time [12]. Unfortunately, such corrections are not readily available for ordinal outcomes in mainstream statistical software. Without small sample bias corrections, re-analyzing the simulated scenarios from Geroldinger et al. [13] using the following GEE-type model (based on Verbeeck et al. [12]):

$$ \begin{aligned} \text{ logit }[P(X_{ikt}\le a|\theta _k) ] &=  \beta _a + \beta _1 G_{ik} + \beta _2 P_k + \sum _{j=3}^5 \beta _j T_{ijkt} + \beta _6 G_{ik}P_k\\    & \quad + \sum _{j=7}^9 \beta _j G_{ik}T_{ijkt} + \sum _{j=10}^{12} \beta _j P_kT_{ijkt}, \end{aligned} $$

where $i=1,2$ is an index for the treatment assignment, $k=1,\dots ,N$ denotes the subjects, $t=1,\dots ,4$ indicates time points, $X_{ikt} \in \{0,1,\dots ,20\}$ are the ordinal VAS scores, $a=1,\dots ,20$, $\theta _{k}$ represents the covariate vector including $G_{ik}$ (treatment group indicator), $P_k$ (period indicator) and $T_{ijkt}$ (discrete time indicator), with independent and heterogeneous autocorrelation structure, shows a slightly liberal type I error: 0.055 (95% CI 0.049; 0.062) and 0.061 (95% CI 0.055; 0.068), respectively. The power to detect the treatment effect (0.27 and 0.26, respectively) is near that of the non-parametric marginal model (0.28), but lower than that of the prioritized unmatched GPC (0.67). This suggests that the conclusions drawn from the comparison between methodologies on the blister outcome [12] can be extended to the ordinal outcome, namely, the prioritized unmatched GPC exhibits higher power to detect treatment effects in longitudinal cross-over trials than GEE-type models in small samples.

However, VAS scores are often criticized for their large inter-subject variability and, to a lesser extent, intra-subject variability (Sect. Pain and pruritus: ordinal or metric visual analogue scale (VAS)). One approach to address this variability is to correct for baseline values by introducing them as covariates in the statistical model. While this is straightforward for regression models, it requires further investigation for non-parametric methods (Sect. Covariate adjustment for non-parametric methods). For the non-parametric marginal model, developing an ANCOVA test capable of handling repeated measures (i.e., the longitudinal profile of the remaining VAS values) is a promising avenue. Furthermore, further research into modeling longitudinal VAS scores could explore pseudo-likelihood models with pairwise fitting [27] or split-sampling techniques [24–26].

### Adaptive designs

When investigating the optimal design for the longitudinal cross-over EBS trial, the mixed-effects model with the highest weight from a model-averaging approach was selected as the “ground truth model” to optimize future studies [15]. Although it is common practice in optimal design to assume a single model as the truth, a potential drawback is that this model may be misspecified, leading to a suboptimal design. This risk can potentially be mitigated by using robust methods like E-family (Bayesian) optimal designs [45, 46], which rely on a distribution of model parameters, rather than fixed values. Alternatively, a model-averaging approach [47, 48] can be employed, wherein multiple models and/or parameters are weighted and incorporated into the optimal design calculations. However, robust model-averaging design methods are in general computationally more time-consuming and they still provide a design that does not adapt as additional information becomes available. Adaptive designs offer a potential solution to this limitation, where the model is updated when new data is collected and the design is adjusted to reflect these updates [49]. This adaptation can occur at both the population level (updating the design for the entire study population) or at the individual level (updating the design for the individual patient), using the initial model as prior information. Future research on optimal study designs could include adaptive elements and investigate the potential gain in information and determine whether adaptive designs are more robust to misspecification of the initial model.

### Individual patient predictions

For longitudinal data, the benefit of having established a mixed-effects models, such as for the treatment of blister lesion counts [12], is that the model can be used for designing individual outcomes in a future study and predicting outcomes in clinical practice. If correctly specified, the individual outcomes can be predicted with either no information of the longitudinal outcome (i.e., based on only baseline covariates), or with additional longitudinal outcomes collected during the study. Individualized treatment effects can be estimated initially and refined as more data become available during the course of treatment. This is commonly called precision medicine, where the treatment is tailored towards the individual patient, based on their unique characteristics and/or observations. In statistical terms, this is often referred to as individual maximum a posteriori (MAP) or empirical Bayes estimation (EBE) for non-linear mixed-effect models. It is defined as:

$$\begin{aligned} {\hat{b}}_i = \mathop {\mathrm {arg\,min}}\limits _{b_i} l_i(\beta ,b_i,z_i,\dots ) + \log (\text {pdf}(b_i;\Omega )), \end{aligned}$$

where $l_i$ is the conditional likelihood (−2 log likelihood) given the fixed effects $\beta$, the random effects $b_i$, and the individual characteristics and design parameters $z_i$. The prior distribution is specified as the probability density function (pdf) given $b_i$, assuming $b_i$ follows a multivariate normal distribution with mean 0 and variance $\Omega$, where $\Omega$ denotes the inter-individual variability.

The individual parameters are estimated using $\beta$, ${\hat{b}}_i$, and $z_i$, enabling predictions at an individual level. Note that the MAP estimation can also guide individual-level design adaptations, such as optimizing the timing of observation time(s) to maximize the information gain (see Sect. Adaptive designs). This approach is especially valuable in rare diseases, where patient numbers are typically very low and maximizing the individual information is often essential.

## Reflection on clinical and patient-relevant aspects

Despite the insights gained in evaluating longitudinal outcomes and optimizing the design of clinical trials for rare diseases, many clinical questions still remain regarding the design of rare disease clinical trials in general and those for EB in particular.

One of the key issues in many rare disease clinical trials is identifying optimal endpoints to evaluate clinical and patient-relevant treatment effects and obtain multi-stakeholder consensus on standardized and validated endpoints [50]. Clinical trials in EB have used various endpoints, including the number of blisters, wound healing, pain reduction, and quality of life [51]. However, selecting endpoint(s) that capture a patient’s overall burden best while remaining sufficiently sensitive to treatment effects can be challenging.

To optimize endpoint selection, engaging with regulatory agencies is essential. Endpoints should align to regulatory guidelines, ensuring feasibility, reproducibility, and patient-centeredness. Moreover, scalable, standardized and validated endpoint measurement tools should be available. For example, although many outcomes have been assessed in EBS, the number of blisters assessed by a rater remains one of the most frequently used outcomes, despite its limitations. Indeed, it is subject to uncertainty (Sect. Number of blister lesions) and therefore less reproducible. Regulatory agencies, such as the FDA, favor an investigator global assessment (IGA). However, it is challenging to convert counting blisters or blister areas into a 5-step IGA defining global disease activity as severe (5), moderate, mild, almost clear and clear (1) [52]. In addition, a patient still must visit a health-care facility for blister assessment, which is associated with discomfort and pain for EB patients. Both the blister uncertainty and the patient’s travel burden might be mitigated by exploring remote blister assessment options using digital tools. Such tools may even increase the number of measurements, improve data granularity, and reduce variability in assessment definitions and ultimately enhancing the evaluation of treatment effects. An important aspect of clinical blister assessment, whether manual or digital, is the differentiation between acute and chronic lesions. While both types contribute to the patient’s overall burden, their pathophysiological processes may differ, and treatments may affect them differently.

Similarly, additional research is needed to evaluate whether patient-centered outcomes measures and patient-reported outcomes, such as VAS scores and QoL, are informative for clinical trials (see Sect. Pain and pruritus: ordinal or metric visual analogue scale (VAS)). Moreover, advances in molecular genetics and pathomechanisms, methodology and technology, alongside facilitated approval procedures and funding initiatives in rare diseases have stimulated translational research, which paves the way to personalized precision medicine and broadens therapeutic possibilities. It is therefore important to acknowledge that even within the same disease, inter- and intra-individual pathogenic heterogeneity and the diversity in therapeutic mechanisms of action may hinder identification of a single optimal endpoint. Even if a reliable and valid endpoint can be identified, questions about the external validity or generalizability of the results remain. Practically speaking, any selected endpoint should undergo validation through inter-and intra-reliability testing. Further research, such as, for example, randomized inference methods [53], is required in this area.

Efforts to reduce patient burden in clinical trials could focus on minimizing placebo treatment duration or avoid it entirely. Potentially, patient registries that collect real-world data, could help reduce the need for extended placebo periods.

Another inherent challenge in rare disease clinical trials is patient heterogeneity. Methodological strategies should be developed to stratify patients based on baseline characteristics and distinguish between responders and non-responders to interventions. Specifically for cross-over trial designs, guidelines and strategies should be developed to mitigate cross-over effects, which imply more complicated interpretation of the case trial results [10].

To address these challenges, fostering international collaboration among clinicians, methodologists, statisticians, regulatory agencies, and patient advocacy groups is essential to ensure good progress in rare disease research [51]. Such collaborations, of which EBStatMax is an example, can help to consolidate data, enhance evidence quality, and facilitate translational research.

## Discussion

The EBStatMax project [9] has provided valuable insight into addressing the unique challenges of rare-disease clinical trials, particularly in evaluating longitudinally repeated measures in Epidermolysis bullosa simplex [12–16]. While the results are based on one particular case, they can be readily extended to other indications and rare diseases. The challenges related to study design and analysis are similar across different rare diseases, allowing promising novel methodological approaches developed for one condition to be applied more broadly.

As is often the case, research projects address only a specific set of questions, leaving other equally relevant issues unaddressed. Furthermore, some answers inevitably lead to to new questions. We have summarized the methodological questions that emerged from this project, including those related to outcome measurements, statistical approaches, and clinical aspects. It is clear that optimizing endpoint selection, addressing measurement variability, exploring missing data mechanisms, incorporating covariate adjustments and developing adaptive trial designs are critical steps for improving the quality and efficiency of rare disease research.

Epidermolysis bullosa simplex is a rare and debilitating genetic disorder characterized by skin fragility, leading to painful blisters and wounds [11]. Improving clinical trial design and outcome measurement in EBS research is essential for developing therapies to alleviate the burden of this condition. By addressing challenges related to identifying optimal endpoints, including patient-centered outcome measures, managing patient heterogeneity, leveraging technological advancement, and minimizing patient burden during clinical trials, the EB research community can move toward more effective treatments and improved quality of life for patients [51].

Collaborative efforts among researchers, clinicians, regulatory agencies, and patient advocacy groups will be essential to advance rare disease clinical research and ultimately benefit individuals living with these conditions. Continued research and international cooperation are critical to making meaningful progress in this vital area of healthcare.

## Data Availability

The data that support the findings of this study are available from the corresponding author upon reasonable request. The underlying original data and the simulation code is available in the following public repository in Github: https://github.com/martingeroldinger/Diacerein_study_data.

## References

[1] Nguengang Wakap S, et al. Estimating cumulative point prevalence of rare diseases: analysis of the orphanet database. Eur J Hum Genet. 2020;28:165–73. 10.1038/s41431-019-0508-0. 31527858 10.1038/s41431-019-0508-0 PMC6974615

[2] Orphanet. Orphanet reports series / procedures 2023. https://www.orpha.net/consor/cgi-bin/Education_Home.php?lng=EN. Accessed: 2023-08-30.

[3] Tisdale A, Cutillo C, Nathan R, et al. The ideas initiative: pilot study to assess the impact of rare diseases on patients and healthcare systems. Orphanet J Rare Dis. 2021;16:429. 10.1186/s13023-021-02061-3. 34674728 10.1186/s13023-021-02061-3 PMC8532301

[4] Valcárcel-Nazco C, et al. Health-related quality of life and perceived burden of informal caregivers of patients with rare diseases in selected european countries. Int J Environ Res Public Health. 2022;19:8208. 10.3390/ijerph19138208. 35805867 10.3390/ijerph19138208 PMC9266302

[5] ASTERIX: advances in small trials design for regulatory innovation and excellence 2013. https://www.asterix-fp7.eu/. FP7-HEALTH-2013-INNOVATION-1 Grant-Agreement No. 603160. Accessed: 2023-08-30.

[6] IDEAL: integrated design and analysis of small population group trials 2013. https://www.ideal.rwth-aachen.de/. EU grant agreement no. 602552. Accessed: 2023-08-30.

[7] INSPIRE: innovative methodology for small populations research 2013. https://warwick.ac.uk/fac/sci/med/research/hscience/stats/completedprojects/inspire/. EU grant agreement no. 602144. Accessed: 2023-08-30.

[8] European Joint Programme on Rare Diseases. Internal call for proposals 2019 - clinical trials methodology demonstration projects 2019. https://www.ejprarediseases.org/funded-projects-demonstration/. EU grant agreement no. 825575. Accessed: 2023-08-30.

[9] EBStatMax 2023. https://www.ejprarediseases.org/funded-projects-demonstration. EBStatMax: State-of-the-art statistical design and analysis for maximal success with minimal patient burden in Epidermolysis bullosa trials, H2020 Societal Challenges, Grant agreement no. 825575. Accessed: 2023-08-30.

[10] Wally V, et al. Diacerein orphan drug development for epidermolysis bullosa simplex: a phase 2/3 randomized, placebo-controlled, double-blind clinical trial. J Am Acad Dermatol. 2018;78:892–901. 29410318 10.1016/j.jaad.2018.01.019

[11] Has C, et al. Consensus reclassification of inherited epidermolysis bullosa and other disorders with skin fragility. Br J Dermatol. 2020;183:614–27. 32017015 10.1111/bjd.18921

[12] Verbeeck J, et al. How to analyze continuous and discrete repeated measures in small sample cross-over trials? Biometrics. 2023;79:1–15. 10.1111/biom.13920 37587671

[13] Geroldinger M, Verbeeck J, Thiel KE, Molenberghs G, Bathke AC, Laimer M, Zimmermann G. A neutral comparison of statistical methods for analyzing longitudinally measured ordinal outcomes in rare diseases. Biometr J. 2024;66(1):e2200236. 10.1002/bimj.202200236. 10.1002/bimj.202200236 36890631

[14] Geroldinger M, et al. Statistical recommendations for count, binary, and ordinal data in rare disease cross-over trials. Orphanet J Rare Dis. 2023;18:391. 38115074 10.1186/s13023-023-02990-1 PMC10729462

[15] Nyberg J, Hooker AC, Zimmermann G, Verbeeck J, Geroldinger M, Thiel KE, Molenberghs G, Laimer M, Wally V. Optimizing designs in clinical trials with an application in treatment of Epidermolysis bullosa simplex, a rare genetic skin disease. Comput Stat Data Anal. 2024;199:108015. 10.1016/j.csda.2024.108015.

[16] Verbeeck J, et al. Composite endpoints, including patient reported outcomes, in rare diseases. Orphanet J Rare Dis. 2023;18:262. 37658423 10.1186/s13023-023-02819-x PMC10474650

[17] Heller G, Manuguerra M, Chow R. How to analyze the visual analogue scale: myths, truths and clinical relevance. Scand J Pain. 2016;13:67–75. 28850536 10.1016/j.sjpain.2016.06.012

[18] Noback D, ad Cuellar PC, Lombardi J, Swart E, Rosenwasser M. Evaluating pain in orthopedic patients: can the visual analog scale be used as a long-term outcome instrument? J Pain Relief. 2015;4:182.

[19] Jensen MP, Chen C, Brugger AM. Interpretation of visual analog scale ratings and change scores: a reanalysis of two clinical trials of postoperative pain. J Pain. 2003;4:407–14. 14622683 10.1016/s1526-5900(03)00716-8

[20] Food and Drug Administration (FDA). Benefit-risk assessment in drug regulatory decision-making Draft PDUFA VI Implementation Plan (FY2018-2022) 2018. URL: chrome-extension://efaidnbmnnnibpcajpcglclefindmkaj/ https://www.fda.gov/files/about Benefit-Risk-Assessment-in-Drug-Regulatory-Decision-Making.pdf. Accessed: 2023-08-30.

[21] Ren X, Chen X, Wang W, Seifu Y. Estimand in benefit-risk assessment. J Biopharm Stat. 2023;33:452–65. 36755379 10.1080/10543406.2023.2170396

[22] Bathke A, Harrar S, Madden L. How to compare small multivariate samples using nonparametric tests. Comput Stat Data Anal. 2008;52:4951–65.

[23] Burchett W, Ellis A, Harrar S, Bathke A. Nonparametric inference for multivariate data: the r package npmv. J Stat Softw. 2017;76:1–18.36568334

[24] Iddi S, Molenberghs G. A marginalized model for zero-inflated, overdispersed and correlated count data. Electron J Appl Stat Anal. 2013;6:149–65.

[25] Ivanova A, Molenberghs G, Verbeke G. Mixed model approaches for joint modeling of different types of responses. J Biopharm Stat. 2016;26:601–18. 26098411 10.1080/10543406.2015.1052487

[26] Molenberghs G, Verbeke G. Models for Discrete Longitudinal Data. New York: Springer; 2005.

[27] Fieuws S, Verbeke G. Pairwise fitting of mixed models for the joint modeling of multivariate longitudinal profiles. Biometrics. 2006;62:424–31. 16918906 10.1111/j.1541-0420.2006.00507.x

[28] Ueckert S. Modeling composite assessment data using item response theory. CPT Pharmacometrics Syst Pharmacol. 2018;7:205–18. 10.1002/psp4.12280. 29493119 10.1002/psp4.12280 PMC5915608

[29] O’Brien P. Procedures for comparing samples with multiple endpoints. Biometrics. 1984;40:1079–87. 6534410

[30] Läuter J. Exact t and F tests for analyzing studies with multiple endpoints. Biometrics. 1996;52:964–70.

[31] Friedrich S, Konietschke F, Pauly M. Analysis of multivariate data and repeated measures designs with the R package MANOVA. RM. arXiv preprint arXiv:1801.08002. 2018

[32] Ristl R, Urach S, Rosenkranz G, Posch M. Methods for the analysis of multiple endpoints in small populations: a review. J Biopharm Stat. 2018;29:1–29. 29985752 10.1080/10543406.2018.1489402

[33] Molenberghs G, Kenward M. Missing Data in Clinical Studies. New York: John Wiley & Sons; 2007.

[34] Beunckens C, Sotto C, Molenberghs G. A simulation study comparing weighted estimating equations with multiple imputation based estimating equations for longitudinal binary data. Comput Stat Data Anal. 2008;52:1533–48.

[35] Carpenter JR, Kenward MG, Bartlett JW, Morris TP, Quartagno M, Wood AM. Multiple Imputation and its Application 2e. Wiley; 2023.

[36] Carpenter JR, Roger JH, Kenward MG. Analysis of longitudinal trials with protocol deviation: a framework for relevant, accessible assumptions, and inference via multiple imputation. J Biopharm Stat. 2013;23:1352–71. 24138436 10.1080/10543406.2013.834911

[37] Rubarth K, Sattler P, Zimmermann HG, Konietschke F. Estimation and testing of wilcoxon-mann-whitney effects in factorial clustered data designs. Symmetry. 2022;14:244.

[38] Deltuvaite-Thomas V. Statistical inference using generalised pairwise comparisons in the presence of censored or missing data. Phd Thesis UHasselt 2022

[39] Dong G, Qiu J, Wang D, Vandemeulebroecke M. The stratified win ratio. J Biopharm Stat. 2018;28:778–9. 29172988 10.1080/10543406.2017.1397007

[40] Bathke A, Brunner E. A Nonparametric Alternative to Analysis of Covariance, 109–120. The Netherlands: Elsevier B.V. Amsterdam; 2003.

[41] Fan C, Zhang D. Rank repeated measures analysis of covariance. Commun Stat - Theory Methods. 2017;46:1158–83.

[42] Thas O, Neve JD, Clement L, Ottoy JP. Probabilistic Index Models. J Royal Stat Soc Ser B: Stat Methodol. 2012;74(4):623–71. 10.1111/j.1467-9868.2011.01020.x.

[43] Mao L, Wang T. A class of proportional win-fractions regression models for composite outcomes. Biometrics. 2021;77:1265–75. 32974905 10.1111/biom.13382 PMC7988303

[44] Hedeker D, Gibbons R. A random-effects ordinal regression model for multilevel analysis. Biometrics. 1994;50:933–44. 7787006

[45] Tod M, Rocchisani J. Comparison of ed, eid, and api criteria for the robust optimization of sampling times in pharmacokinetics. J Pharmacokinet Biopharm. 1997;25:515–37. 9561492 10.1023/a:1025701327672

[46] Dodds M, Hooker A, Vicini P. Robust population pharmacokinetic experiment design. J Pharmacokinet Pharmacodyn. 2005;32:33–64. 16205840 10.1007/s10928-005-2102-z

[47] Chatfield C. Model uncertainty, data mining and statistical inference. J Royal Stat Soc Ser A: Stat Soc. 1995;158(3):419–44.

[48] Bretz F, Pinheiro J, Branson M. Combining multiple comparisons and modeling techniques in dose-response studies. Biometrics. 2005;61:738–48. 16135025 10.1111/j.1541-0420.2005.00344.x

[49] Zamuner S, et al. Adaptive-optimal design in pet occupancy studies. Clinical Pharmacol Therapeutics. 2010;87:563–71. 10.1038/clpt.2010.9 20336064

[50] Korte E, et al. Towards a roadmap for COSEB: the next steps in harmonization of outcomes for epidermolysis bullosa. Br J Dermatol. 2024;191:463–5. 10.1093/bjd/ljae200. 38865426 10.1093/bjd/ljae200

[51] Korte E, et al. Heterogeneity of reported outcomes in epidermolysis bullosa clinical research: a scoping review as a first step towards outcome harmonization. Br J Dermatol. 2023;189:80–90. 37098154 10.1093/bjd/ljad077

[52] Bauer JW, et al. Use of an investigator’s global assessment scale to evaluate disease severity in patients swith epidermolysis bullosa simplex. SKIN J Cutaneous Med. 2018;2:S. 10.25251/skin.2.supp.55.

[53] Kennes L, Cramer E, Hilgers R-D, Heussen N. The impact of selection bias on test decisions in randomized clinical trials. Statist Med. 2011;30:2573–81. 10.1002/sim.4279 21717489

